# The Perception of Time Is Underestimated in Adolescents With Anorexia Nervosa

**DOI:** 10.3389/fpsyt.2018.00121

**Published:** 2018-04-09

**Authors:** Carmelo M. Vicario, Kim Felmingham

**Affiliations:** ^1^School of Psychology, University of Tasmania, Hobart, TAS, Australia; ^2^Dipartimento di Scienze Cognitive, Psicologiche, Pedagogiche e degli Studi Culturali, Messina, Italy; ^3^Department of Psychology and Neurosciences Leibniz Research Center for Working Environment and Human Factors, Dortmund, Germany; ^4^School of Psychological Sciences, University of Melbourne, Parkville, VIC, Australia

**Keywords:** anorexia nervosa, time processing, time underestimation, long-term restrictive diet, symptomatic trait

## Abstract

Research has revealed reduced temporal discounting (i.e., increased capacity to delay reward) and altered interoceptive awareness in anorexia nervosa (AN). In line with the research linking temporal underestimation with a reduced tendency to devalue a reward and reduced interoceptive awareness, we tested the hypothesis that time duration might be underestimated in AN. Our findings revealed that patients with AN displayed lower timing accuracy in the form of timing underestimation compared with controls. These results were not predicted by clinical, demographic factors, attention, and working memory performance of the participants. The evidence of a temporal underestimation bias in AN might be clinically relevant to explain their abnormal motivation in pursuing a long-term restrictive diet, in line with the evidence that increasing the subjective temporal proximity of remote future goals can boost motivation and the actual behavior to reach them.

## Introduction

Anorexia nervosa (AN) is a disorder of unknown etiology, characterized by severe eating restriction and distorted body image (1), which mainly affects young women (2). The estimated incidence of this disorder in the population is around 8 per 100,000 persons per year (3), while the mortality rate is the highest of any psychiatric disorder (4).

An excessive self-control linked to reward processing is considered one hallmark symptom in AN [e.g., Ref. (5–7)]. This is also suggested by recent investigations (8–10) using temporal discounting paradigms referring to a monetary reward (i.e., participants were asked to choose between smaller-sooner and larger-later monetary rewards) that examine the level to which a reward is devalued (discounted) over time (11). In particular, this research has revealed a lower devaluing associated with delayed reward in AN compared with healthy controls AN had significantly lower discount rates (i.e., less steep discounting) in the intertemporal choice task (10). In other words, as explained by Steinglass et al. (8), one dollar in 3 months was worth more for the AN group than it was for the healthy controls group.

Such reduced temporal discounting in AN has been interpreted as proof of enhanced ability (thus higher self-control) to delay a reward, which might help to explain their capacity to maintain a food restriction for a long time, possibly in favor of a future, more attractive reward—i.e., a further weight loss (8). However, the literature is not consistent as no temporal discounting difference between AN and controls has been documented by others [i.e., Ref. (11–13)]. These contrasting results have been explained by methodological discrepancies in task design and/or age of participants (12, 13).

In research on temporal discounting in healthy populations, several scientists have suggested the importance of separating the perception of the value associated with a reward from the perception of a delay in temporal discounting [e.g., Ref. (14–17)]. In support of this suggestion, a study by Kim and Zauberman (18) has shown that the level of overall time contraction (i.e., how long or short individuals perceive time horizons to be overall) contributes to the degree of hyperbolic discounting—i.e., the tendency to choose a smaller-sooner reward over a larger-later reward. These authors found that the individual levels of hyperbolic discounting were positively correlated with the participants’ time estimation length, i.e., the longer the time estimation, the higher the tendency to choose a smaller-sooner reward over a larger-later reward. This result has been confirmed in a subsequent work showing that people who overestimate the passage of time hold less value in delayed rewards (19). Moreover, a study by Suo et al. (20) found lower choice percentage for the smaller-immediate reward in participants who tended to underestimate time, compared with participants who tended to overestimate time. Taken together, the evidence of a link between temporal discounting rate and the subjective experience of time suggests a specific prediction about the perception of time in AN. In particular, one might expect a tendency of individuals with AN to underestimate the duration of temporal intervals, as an effect of a reduced temporal discounting in this clinical population (8–10, 21).

Dysfunction in the ability to perceive time duration in individuals with AN can also be predicted in relation to their altered interoceptive functions [e.g., Ref. (22)], in line with evidence that time perception is modulated by interoception [e.g., Ref. (23–25)]. Meissner and Wittmann (26) have found that time estimation accuracy correlates with both the slope of cardiac slowing during the perception of temporal intervals and the conscious awareness of an individual’s own heart beats. Moreover, Di Lernia et al. (27) have recently found a temporal underestimation of the duration of interoceptive stimuli in relation to a diminished processing of high salience stimuli from the body. This latter work further supports the prediction of a temporal underestimation in AN, given the evidence of altered processing of interoceptive (22) and body-related (28) information in this clinical population.

The hypothesis that AN might be associated with dysfunctional time estimation is also supported by the neuroimaging literature. Structural and functional data have shown that the frontostriatal pathway, which is known to play a key role in the experience of time [e.g., Ref. (29–33)], is the most susceptible neural structure to cognitively maintained restraint of appetite in AN [Ref. (34), see also Ref. (35), for a review]. The insula is another region considered putatively important for explaining the pathophysiology of individuals with AN [Ref. (32, 33, 35–38) for a review], and the research has demonstrated its direct involvement in time keeping functions [e.g., Ref. (39–41); see Ref. (42) for a review]. The neural factors predictive of potential time keeping alterations in AN include dysfunction in the dopaminergic system, which is also directly implied in the regulation of the mental clock’s beats [e.g., Ref. (30, 43–46)]. Evidence of dysregulation in dopaminergic processes in AN is provided by a PET study in subjects who recovered from AN (47). These authors found increased dopamine (i.e., D2/D3) receptor (DRD3) binding in the ventral striatum, a region that modulates responses to reward stimuli (48) and the subjective experience of time (49, 50). Moreover, research suggests a greater dopamine model reward-learning signal in the anteroventral striatum, insula, and in the prefrontal cortex of AN patients (51).

From a clinical point of view, the evidence of a timing alteration in individuals with AN might be helpful in explaining the dietary restriction associated with AN. A time underestimation bias might contribute to the abnormal motivation of AN in delaying or skipping the consumption of a meal (a primary reward) for a longer-term more desirable reward (i.e., the weight loss). In presence of such a bias, individuals with AN might mentally represent their main goal (i.e., losing more weight) as more temporally proximal than how it is in reality. This could explain their strong motivation in keeping a long-term restrictive diet. An alternative, not mutually exclusive, suggestion is that a temporal underestimation might cause a misperception of meal duration and between-meal epochs. In this regard, one might assume that if between-meal intervals seem shorter than they actually are, then the motivation for the next meal is reduced. Evidence in support for such arguments exists in research on obesity (e.g., people eat more often when the interval between meals seem longer), smoking, and technology use (52–55). Interestingly, the use of a temporal discounting paradigm in previous studies in AN did not allow clarifying whether the reduced discounting response selectively reflect the value of the delayed reward, or if it might also be linked to the encoding of temporal information emerging from the execution of such a paradigm. Therefore, testing AN with an explicit timing task might also address this timely question.

Based on this literature, in this study, we tested if there was a time processing deficit in AN by using a supra-second time estimation task of visual stimuli. We also investigated any role played by attention and working memory (WM) skills, which are known to be predictive of temporal performance in healthy humans and clinical populations (30, 56–62). Moreover, we explored the contribution of depression, stress, and anxiety symptoms, which might influence time keeping skills [e.g., Ref. (63)].

## Participants

Data from individuals with AN and healthy controls were extracted from the Brain Resource International Database (BRID^1^). This database contains data from multiple laboratories (New York, Rhode Island, Nijmegen, London, Adelaide, and Sydney) that have been acquired using standardized data acquisition techniques for cognitive tasks (IntegNeuro) including the time estimation task. Inter-lab reliability and test–retest reliability measures are high as documented in previous works [e.g., Ref. (64–66)]. The request of data is only granted to scientists who are formally registered to BRID. Access to the database was approved after the evaluation of our research proposal on time perception in AN. The review of our proposal was executed by other colleagues formally registered to BRID. After formal approval, the manager of the database provided an excel copy of all the available data on AN and control participants.

The AN participants were recruited from two adolescent inpatient eating disorder programs at associated teaching hospitals of the University of Sydney, Australia (The Children’s Hospital at Westmead and Westmead Hospital). Specific inclusion criteria for AN participants included DSM-IV diagnosis of AN, female, aged between 12.5 and 17.5 years and in their first hospital admission at the time of recruitment. The exclusion criteria of this database included a personal or family history of mental illness, brain injury, neurological disorder, serious medical condition, drug/alcohol addiction, first-degree relative with bipolar disorder, schizophrenia, or genetic disorder, no history of bulimia nervosa, or BMI above 17.5 on day of baseline assessment. We adopted the 10th percentile of the body mass index as a cutoff for underweight in the control group (67). From two initial samples of 41 patients and 41 controls, we excluded participants with less than 8 years of education, to make this variable equivalent between the two samples. Furthermore, we did not include participants with missing information with regard to BMI, and participants providing outlier performance (i.e., ±3 SD). Therefore, the final analysis included a sample of 30 AN patients (mean age = 15.43 ± 1.60 SD) and 21 healthy controls (mean age = 15.32 ± 1.81). All participants gave written informed consent to participate in the study. The study was approved by the University of Tasmania, School of Psychology, Research Ethics Committee.

## Method

### Psychometric Measures

Anorexia nervosa diagnosis was made using the clinician-administered DSM-IV criteria (2). To estimate disorder severity and to assess for eating disorder-related psychopathology, individuals completed the Eating Disorders Inventory-3 (68). WM and attention performance were examined by using, respectively, the *Digit Span* (69) and the *Switching of Attention* (70) tasks. To assess for depressed, anxious, and stressed mood, the depression anxiety and stress scale (DASS) was administered (71). A detailed description of the psychometric measures is provided in the following paragraphs.

## Tasks and Procedure

Participants were seated in a sound attenuated room in front of a touchscreen computer (NEC MultiSync LCD 1530V). All participants completed the cognitive tests as part of a reliable and valid computerized test battery (64, 66). Tests were administered using prerecorded task instructions (*via* headphones) and computerized and voice recording was used for answers. All participants performed a practice trial before the formal completion of the proposed tasks. Responses were provided by using the touchscreen.

### Digit Span Task

Participants were presented with a series of digits on the touchscreen, separated by a 1-s interval. The subjects were then immediately asked to enter the digits on a numeric keypad on the touchscreen. In the first part of the test, subjects were required to recall the digits in forward order and reverse order in the second. In each part, the number of digits in each sequence was gradually increased from 3 to 9, with two sequences at each level. The dependent measure was the total number of correct trials forward and backward.

### Switching of Attention Task

This modified version of the Trail Making Test consisted of two parts. The first, a measure of psychomotor speed, required the connecting of numbers in ascending sequence (i.e., 1-2-3-, etc.) (Switching of Attention − Number). The second, requiring speeded cognitive flexibility, asked participants to connect numbers and letters in an ascending but alternating sequence (i.e., 1-A-2-B, etc.) (Switching of Attention − Number/Letter). Time for completion for each part served as dependent variables.

### The Time Estimation Task

**Figure 1:**
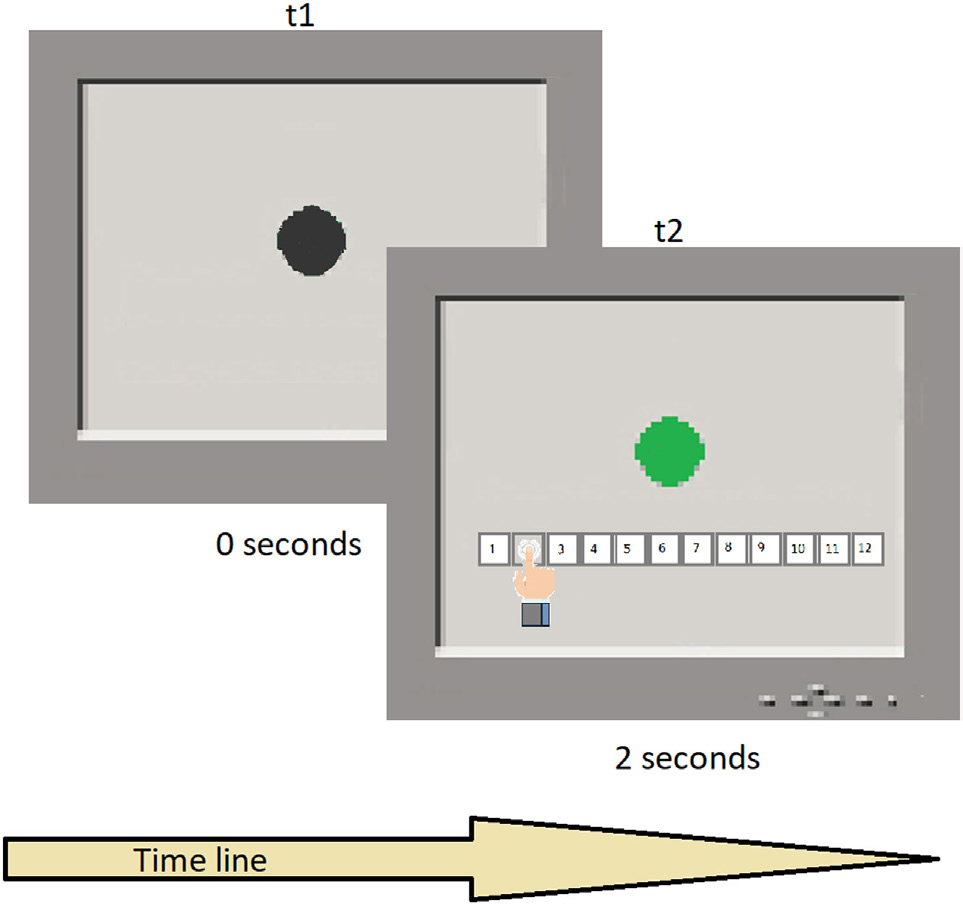
Typical trial sequence. t1 shows the black circle presentation in the screen; t2 shows the color switch after a temporal interval and the participant’s response.

A black circle appeared on the screen, turning green for times varying between 1 and 12 s, in steps of 1 s, in pseudo-random order and for a total of 12 intervals. Therefore, the number of trials was 12. Each participant was required to attend to the screen and estimate the duration of the target trace on the screen, using keys on a fixed display touchpad at the bottom of the screen with the duration range between 1 and 12 s. Each temporal switch was presented once. Task duration was approximately 3 min (see Figure 1 for a schematic representation of the task execution). The task assesses the ability to estimate time intervals without a clock and relates to the ability to pre-plan actions, decide temporal onset, monitor the time course of initiation, and anticipate outcomes. For more information about the task, refer to Block et al. (72) and Gunstad et al. (73).

## Data Analysis

Participants’ task performance was evaluated by considering the *proportional bias* (PB), which provides a measure of the estimation accuracy calculated from the 12 temporal intervals, where the bias for each trial is calculated as a positive or negative percentage of the actual presented interval. PB is estimated from the absolute value of the average difference between the actual duration of the stimulus and the user-estimated duration, weighted by the length of the stimulus. Thus, an overall positive score (i.e., >0) indicates a temporal overestimation; while an overall negative score (i.e., <0) indicates a temporal underestimation. As *post hoc* analysis, we also calculated the *estimation bias variability* (EBV), which represents the SD average of the PB. Timing variability measure reflects how repeated responses are scattered from their target within a particular experimental condition. We have calculated this parameter as a further exploratory measure, with the purpose to explore participants’ variability in detecting the duration of temporal intervals.

Proportional bias and EBV scores of our clinical and control samples were compared by using pairwise *t*-test comparisons. Although we were interested in exploring any role of other variables on time keeping skills, we choose the *t*-test comparison, instead of the analysis of covariance (ANCOVA), in keeping with the suggestion of a recent work (74) that discourages the use of ANCOVA in the case of classification designs, that is designs that indicate a comparison of different populations such as in our case. The participants PB scores were also compared against the 0 score *via* one sample *t*-test, to investigate if the timing performance of our two samples significantly deviated from the 0 score, which reflects the timing performance in absence of biases. Finally, *Pearson* correlation analyses were implemented to measure any relationship between the time estimation performance and the cognitive/affective measures collected for our participants. We run separate analyses for AN and controls as the intent of our research was exploring any between groups difference with regard to the several variables included in our research.

The *p* value was adjusted with Bonferroni correction, as PB and EBV scores were compared with 14 variables (75). Therefore, the corresponding level of significance for the correlation outputs is <0.003 (i.e., 0.05/14, see Table 2 for details). Data analysis was performed using Statistica software, version 8.0, Stat Soft, Inc., Tulsa, OK, USA.

## Results

The sample size was sufficiently large (i.e., ≥46) for the related effect size of Cohen’s *d* = 0.75, a statistical power of 0.8 and a probability level of 0.05. The observed *post hoc* statistical power was 0.833. All these analyses were implemented via *Free Statistics Calculator*.^2^ Table 1 presents a summary of means and SDs on demographic, cognitive, and clinical variables for the groups and test statistics for between group comparisons (i.e., pairwise *t*-test). A significant between groups difference was reported for all clinical measures and the BMI score, while no difference was reported for cognitive and demographic variables (Table 1 for details).

**Table 1:** The table reports the means and the *t*-test scores for the examined clinical and cognitive affective performance associated to AN and control participants.

	Mean—AN	Mean—controls	*t*-Test	*p*-Level
Age	M = 15.43, SD = 1.66	M = 15.33, SD = 1.81	0.217	0.828
Education	M = 10.63, SD = 1.62	M = 10, SD = 1.70	1.341	0.186
Depress	M = 9.72, SD = 5.90	M = 2.47, SD = 2.85	4.904*	<0.001
Anxiety	M = 5.88, SD = 4.12	M = 1.23, SD = 1.17	4.871*	<0.001
Stress	M = 8.52, SD = 4.98	M = 3.42, SD = 3.12	3.810*	<0.001
Digitot	M = 6.25, SD = 2.90	M = 6.70, SD = 2.16	0.228	0.820
Digitsp	M = 5.74, SD = 1.60	M = 5.92, SD = 1.32	0.261	0.794
Rdigitot	M = 4.51, SD = 2.37	M = 3.88, SD = 2.29	1.638	0.107
Rdigitsp	M = 4.71, SD = 1.91	M = 4.40, SD = 1.52	1.505	0.138
Swoadur1	M = 18,656, SD = 4,260	M = 19,652, SD = 4,155	−1.799	0.081
Swoaerr1	M = 0.80, SD = 1.28	M = 0.66, SD = 0.960	0.240	0.811
Esoadur2	M = 40,276.1, SD = 13,273.2	M = 41,076.1, SD = 13,057	−1.070	0.289
Esoaerr2	M = 1.02, SD = 2.00	M = 1.29, SD = 1.56	−0.872	0.387
BMI	M = 16.10, SD = 1.07	M = 21.11, SD = 4.01	−6.485*	<0.001

*A significant result.

*Digitot (digit span forward, correct trials); Digitsp (digit span forward, recall span); Rdigitot (digit span reverse, correct trials); Rdigitsp (digit span reverse, recall span); Swoadur1 (switching of attention, completion time—digits); Swoaerr1 (switching of attention, errors—digits); Esoadur2 (switching of attention, completion time—digits + letters); Esoaerr2 (switching of attention, errors—digits + letters); BMI (body mass index)*.

**Figure 2:**
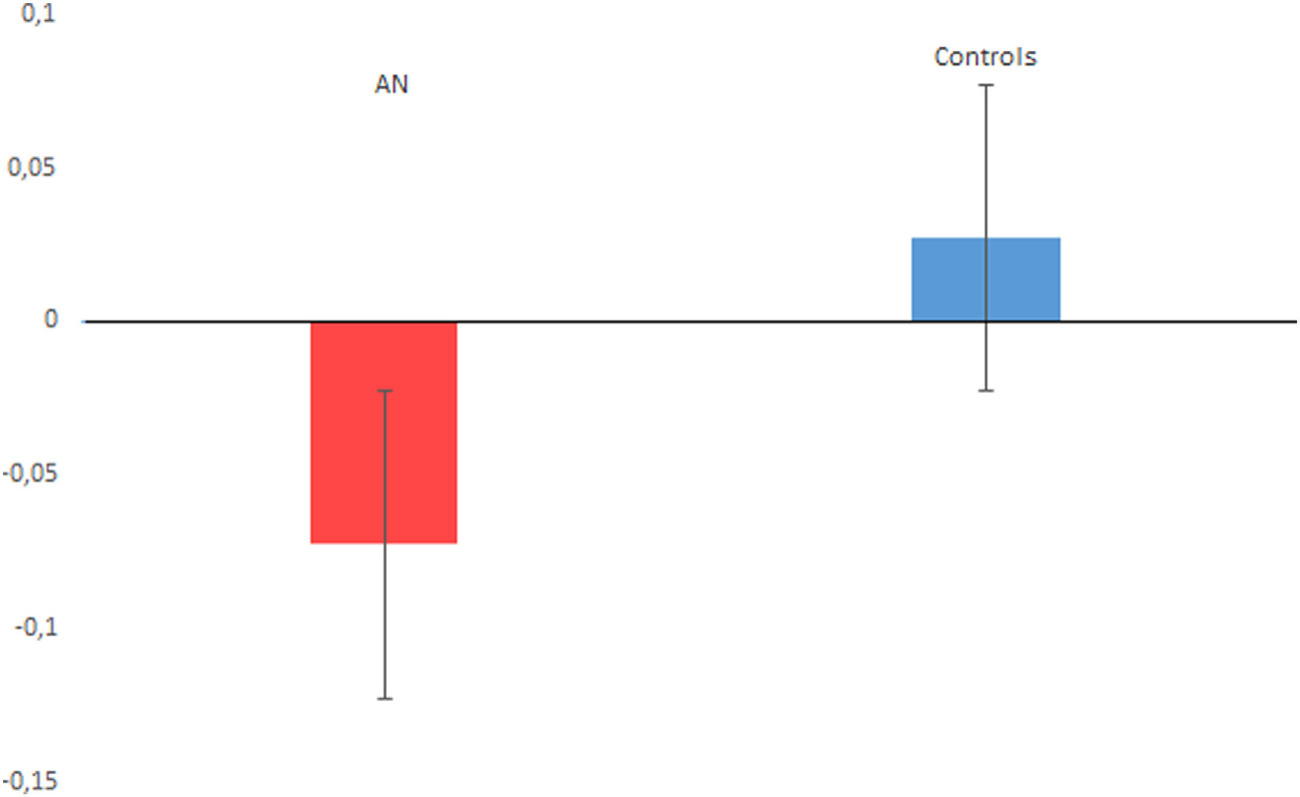
The figure plots the proportional bias performance associated with anorexia nervosa (AN) and control participants. A negative value indicates time underestimation, while a positive value indicates time overestimation. Vertical bars denote ± SEs.

The *t*-test on timing performance documented a significant between group difference for the PB score, which was lower for the AN sample (M = −0.072, SD = 0.102), compared with controls (M = 0.027, SD = 0.152) (*t* = −2.80, *p* = 0.007, Cohen’s *d* = 0.75), see Figure 2 for details. The one sample *t*-test relative to 0 revealed a significant difference for the AN sample (*t* = −3.890, *p* < 0.001, Cohen’s *d* = 0.69). By contrast, no significant difference from the 0 score was reported comparing the performance of the control group (*t* = 0.817, *p* = 0.423, Cohen’s *d* = 0.20). Finally, we did not find a significant difference for the EBV score (*p* = 0.121).

The correlation analyses of AN and control participants did not reveal significant results according the adjusted *p*-level (see Table 2 for details).

**Table 2:** The table provides details about the correlation results between the PB and estimation bias variability (EBV) scores of the AN and the control groups and their performance with regard to clinical and cognitive measures.

	PB *AN*		EBV *AN*		PB *controls*		EBV *controls*	
	*R*	*p*-Level	*R*	*p*-Level	*R*	*p*-Level	*R*	*p*-Level
Age	−0.289	0.120	−0.114	0.546	−0.202	0.378	−0.0381	0.869
Education	−0.306	0.100	−0.063	0.738	−0.270	0.236	−0.117	0.611
Depress	−0.135	0.476	−0.052	0.782	−0.273	0.230	−0.234	0.307
Anxiety	−0.102	0.588	0.054	0.775	−0.035	0.880	−0.023	0.921
Stress	−0.059	0.754	−0.011	0.952	−0.120	0.601	−0.204	0.373
Digitot	0.397	0.032	−0.460	0.012	−0.181	0.430	0.292	0.198
Digitsp	0.427	0.020	−0.495	0.006	−0.361	0.107	0.142	0.537
Rdigitot	0.167	0.385	−0.290	0.126	−0.298	0.188	0.357	0.111
Rdigitsp	0.157	0.413	−0.292	0.123	−0.293	0.197	0.257	0.259
Swoadur1	0.171	0.365	0.001	0.999	0.018	0.9350	−0.072	0.754
Swoaerr1	0.099	0.601	0.271	0.146	0.158	0.493	0.314	0.165
Esoadur2	−0.024	0.898	0.472	0.008	0.217	0.343	−0.023	0.919
Esoaerr2	0.312	0.092	0.427	0.018	0.3100	0.170	0.191	0.406
BMI	0.053	0.779	0.019	0.919	−0.304	0.178	0.075	0.746

*Digitot (digit span forward, correct trials); Digitsp (digit span forward, recall span); Rdigitot (digit span reverse, correct trials); Rdigitsp (digit span reverse, recall span); Swoadur1 (switching of attention, completion time—digits); Swoaerr1 (switching of attention, errors—digits); Esoadur2 (switching of attention, completion time—digits + letters); Esoaerr2 (switching of attention, errors—digits + letters); BMI (body mass index)*.

## Discussion

In this study, we provided evidence that time perception is underestimated in AN. This result is in agreement with our initial hypothesis based on the research documenting temporal underestimation in the presence of reduced temporal discounting (20) and altered processing of interoceptive/body information (27), which have been documented in AN [e.g., Ref. (8, 10, 22, 28)]. Moreover, the timing performance of the AN group significantly deviated from the 0 score of the PB measure, where the 0 score represents the timing performance in absence of any bias. Therefore, the AN timing performance can also be described as being less accurate than that of the control sample. This result can be explained in relation to the reduced cardiac awareness in AN (76, 77). In fact, the lower the awareness of individual heart beats, the lower the time estimation accuracy (26). However, we did not measure cardiac awareness in our AN participants, and therefore this suggestion remains to be directly tested in future works. As outlined in the Section “Introduction,” a possible implication of a temporal underestimation bias in AN is that these patients might perceive their forthcoming goals—such as losing more weight—as more temporally proximal than how they are in the reality. This might contribute to explaining their heightened motivation in keeping a long-term restrictive diet. Such an interpretation is in line with the evidence (78) that increasing the subjective temporal proximity of remote future goals can boost motivation and the actual behavior to reach them, regardless of whether these goals are described in mildly or highly pessimistic terms. In keeping with this interpretation, we suggest that the time underestimation pattern reported in AN might be interpreted as the sign of a *temporal proximity bias*, which might affect their decisions for perspective goals (i.e., the weight loss) or outcomes they are interested in. Importantly, the absence of any correlation with the examined clinical, cognitive, and demographic measures corroborates the suggestion that the AN timing alteration documented in our study might be a symptomatic trait of this psychiatric condition, rather than being the epiphenomenon of other related deficits. Moreover, the evidence of a temporal underestimation in AN suggests that the reduced temporal discounting reported in previous work [e.g., Ref. (8)] might not be a hallmark of this clinical population, but a secondary effect of such timing bias. This is in line with previous evidence in healthy humans [e.g., Ref. (20)] reporting lower choice percentage for the smaller-immediate reward in participants who tended to underestimate time.

There are limitations in this study that should be mentioned. No information about the sub-type of AN condition (*restrictive* or *purging*) was available from the database. This would have been relevant in terms of exploring the possible contribution of impulsivity, as this is known to differ between the two subtypes of AN (79) and can affect timing performance (80). The timing task adopted in our current study did not include AN disorder-specific stimuli (i.e., picture of food stimuli) and/or other reward-related conditions. This would be another interesting aspect to explore in future investigations, as it might clarify whether and how the exposure to a primary reward, which may be perceived as an aversive experience in AN (81, 82), affects time processing. We anticipate that an overestimation response might be predicted in the timing performance of AN in the presence of food stimuli, in line with the evidence of temporal overestimation in response to negative, stressful/fearful outcomes and anxiety [e.g., Ref. (24, 83–85)]. The absence of measures to establish the temporal discounting response and the interoceptive awareness in our AN participants do not allow us to verify whether the reported temporal underestimation pattern is directly related with these measures, as it can be inferred from previous works in this field [e.g., Ref. (20, 26)]. Therefore, the role of temporal discounting and interoception need to be explicitly tested in future investigations.

Strengths of our study are represented by the potential theoretical and clinical implications of the current discovery for the interpretation of AN disorder; the rigid inclusion/exclusion criteria; and the examination of demographic, clinical, and cognitive measures for the interpretation of the reported timing pattern.

## Conclusion

The results of our study add new insights for the clinical interpretation of AN, suggesting that the temporal underestimation might contribute to the restrictive dietary pattern of this psychiatric disorder. In particular, we suggest that, because of a temporal underestimation bias, AN participants might perceive their future goals (i.e., the loss of further weight) as more proximal. This might contribute to the origin of their extreme motivation in pursuing a restrictive diet for a long time, as increasing the subjective temporal proximity of remote future goals can boost motivation and the actual behavior to reach them (78).

The absence of relationships between cognitive (WM and attention) and timing performance also suggests that the temporal underestimation bias of AN might be a symptomatic trait of this clinical condition. Moreover, the absence of such relationships, together with the absence of significant WM and attention deficits in this AN group, provide insights with regard to the possible neural underpinning of the reported timing alteration. In particular, one might speculate a main role of the insula cortex, over other neural regions, which activity is known to be altered in AN [Ref. (35), for a review]. This suggestion is provided in line with a recent study (86) which has dissociated the role of the insula activity in time processing from the role of basal ganglia for WM and attention processing. The potentially central role of the insula in the timing alteration of AN might make sense also of the food restriction habit of these patients, given the role of this region in regulating hunger/satiety signaling (87, 88).

Future investigations are required to extend this research in adult AN participants. The inclusion of other timing paradigms such as temporal expectation tasks [e.g., see Ref. (89–91), for a review], might allow us to more directly test the hypothesis that AN is affected by a *temporal proximity bias* for forthcoming goals.

## Ethics Statement

All participants gave written informed consent during their recruitment. The study was approved by the Western Sydney Area Health Services Human Research Ethics Committee and by the University of Tasmania, School of Psychology, Research Ethics Committee.

## Author Contributions

Study concept and design, statistical analysis and interpretation of data, and critical revision of the manuscript: CV and KF. Acquisition of data and drafting of the manuscript: CV. All authors read and approved the version to be published.

## Conflict of Interest Statement

The authors declare that the research was conducted in the absence of any commercial or financial relationships that could be construed as a potential conflict of interest.

## Footnotes

^1^http://www.brainnet.net/about/governance-and-management/.

^2^https://www.danielsoper.com/statcalc/calculator.aspx?id=49.
